# Managing Arterial Hypertension in Chronic Renal Failure: Myths, Mechanisms, and Therapeutic Realities

**DOI:** 10.3390/jcm15031250

**Published:** 2026-02-04

**Authors:** Francesco Versaci, Domenico Maria Giamundo, Giacomo Frati, Lucia Fatima Di Napoli, Giuseppe Biondi-Zoccai, Edoardo Roberto Ginghina

**Affiliations:** 1 Department of Cardiology, Santa Maria Goretti, 04100 Latina, LT, Italy; francesco.versaci@ausl.latina.it; 2 Department of Medical-Surgical Sciences and Biotechnologies, Sapienza University of Rome, 04100 Latina, LT, Italy; dmgiamundo@policlinicocasilino.it (D.M.G.); giacomo.frati@uniroma1.it (G.F.); dinapoli.2004978@studenti.uniroma1.it (L.F.D.N.); edoardoroberto.ginghina@uniroma1.it (E.R.G.); 3 IRCCS NEUROMED, 86077 Pozzilli, IS, Italy; 4 Maria Cecilia Hospital, GVM Care & Research, 48033 Cotignola, RA, Italy

**Keywords:** hypertension, long-term follow-up, renal denervation, renal failure

## Abstract

Hypertension is highly prevalent among patients with chronic kidney disease (CKD), contributing significantly to cardiovascular morbidity and progressive renal decline. This overview explores the intricate pathophysiologic mechanisms driving hypertension in renal insufficiency, including volume overload, renin–angiotensin–aldosterone system (RAAS) activation, sympathetic overactivity, and vascular dysfunction. Diagnostic challenges such as white-coat hypertension and the underuse of ambulatory monitoring are discussed, along with the importance of volume assessment and target organ evaluation. We also emphasize individualized management strategies combining lifestyle modification, pharmacotherapy—including RAAS inhibitors, diuretics, and novel agents—and the growing role of device-based interventions. In particular, renal denervation (RDN) has emerged as a potential adjunctive option for selected patients with resistant hypertension in CKD, with preliminary evidence suggesting blood pressure reduction in selected and carefully studied populations, including dialysis-dependent patients. Special considerations for transplant recipients, elderly individuals, and those on dialysis are highlighted, underscoring the need for nuanced, patient-centered care. Misconceptions surrounding RAAS blockade, dialysis hypotension, and therapeutic inertia are critically appraised. Finally, future directions point to biomarker-driven approaches, digital health integration, and large-scale trials on RDN to refine treatment paradigms. This comprehensive synthesis offers a pragmatic framework for clinicians managing hypertension in CKD, aligning mechanistic insights with emerging evidence and clinical realities.

## 1. Introduction

Hypertension is a nearly ubiquitous comorbidity in patients with chronic kidney disease (CKD), affecting the majority of individuals across all stages of renal impairment [1]. Raised blood pressure (BP) reflects underlying pathophysiologic disturbances but also creates a self-perpetuating cycle by accelerating the progression of renal dysfunction and substantially increasing cardiovascular risk [2]. Indeed, even modest elevations in systolic blood pressure (SBP) have been clearly associated with faster declines in estimated glomerular filtration rate (eGFR), increased left ventricular hypertrophy, and greater incidence of heart failure and stroke [3,4]. This bidirectional relationship forms a complex and self-perpetuating cycle that demands nuanced clinical management, notwithstanding the limitations of current clinical models [5].

Despite the high prevalence and clinical importance of hypertension in CKD, control rates remain suboptimal and indeed resistant hypertension—defined as BP above goal despite three or more antihypertensive agents, including a diuretic—is disproportionately common in this population [6], with key challenges primarily arising from altered drug pharmacokinetics, volume overload, neurohormonal activation, and diagnostic pitfalls such as white-coat hypertension or pseudohypertension [7]. Furthermore, therapeutic inertia, reluctance to escalate renin–angiotensin–aldosterone system (RAAS) blockade in advanced CKD, and misconceptions about hemodynamic stability in dialysis patients compound the difficulty of effective BP management [8].

This review aims to critically examine the major pathophysiologic mechanisms underlying hypertension in renal insufficiency, examine current evidence for pharmacologic and device-based treatment options, and confront widespread myths that hinder optimal care. We place special emphasis on the evolving role of renal denervation (RDN) and the importance of tailoring strategies to diverse clinical scenarios, including dialysis and transplant settings [9,10]. Our goal is to provide clinicians with a clear, evidence-informed framework for managing hypertension in CKD—one that integrates mechanistic understanding, critical appraisal of therapeutic efficacy, and pragmatic consideration of patient-centered outcomes.

## 2. Umbrella Review on Hypertension in Chronic Kidney Disease

This manuscript is primarily conceived as a narrative review. However, to contextualize key areas of uncertainty and evidence gaps, we also performed a focused umbrella overview of published systematic reviews addressing hypertension in chronic kidney disease.

The aim of this approach was not to derive pooled estimates for clinical decision-making, but to provide a high-level synthesis of existing secondary evidence and to highlight areas of consistency and limitation across reviews, in line with methodological considerations previously described for umbrella reviews [11].

Accordingly, PubMed (https://pubmed.ncbi.nlm.nih.gov/?term=hypertension+AND+(renal+failure+OR+nephropathy+OR+ESRD)+AND+systematic%5bsb%5d) was queried on 29 December 2025, using the following search string: hypertension AND (‘renal failure’ OR nephropathy OR ESRD) AND systematic[sb]. From a total of 1149 records, five systematic reviews were included: three focused on dialysis populations and two addressing blood pressure–kidney relationships outside strict ESRD cohorts [12,13,14]. The main characteristics, findings, and limitations of the included systematic reviews are summarized in Table 1.

The dialysis-focused reviews examined intradialytic hypertension and hypotension, antihypertensive strategies, and proposed blood pressure targets, including one meta-analysis restricted to randomized controlled trials [13,15].

Across these reviews, intradialytic blood pressure instability was consistently associated with adverse cardiovascular outcomes and increased mortality risk, although the evidence was predominantly observational. Definitions of intradialytic hypertension and hypotension, timing of measurements, dialysis prescriptions, and adjustment for volume status varied substantially, limiting comparability and leaving room for residual confounding and reverse causation.

The randomized-trial meta-analysis suggested that blood pressure-lowering interventions in hypertensive hemodialysis patients were associated with reduced all-cause mortality, with pooled estimates favoring active treatment [13]. However, the small number of trials, heterogeneity in patient populations and drug classes, and incomplete reporting of methodological details preclude firm conclusions regarding universal blood pressure targets, and proposed systolic values below approximately 140 mmHg should be interpreted as hypothesis-generating rather than definitive.

Complementary qualitative synthesis emphasized that blood pressure control in renal failure is not purely pharmacologic but closely linked to fluid management and dialysis delivery. Dry-weight optimization, sodium balance, and careful timing or withholding of antihypertensive medications were highlighted as key strategies to prevent intradialytic hypotension, while acknowledging the absence of robust evidence for a single “ideal” intradialytic or interdialytic blood pressure range.

In non-dialysis chronic kidney disease, a systematic review and meta-analysis on Dietary Approaches to Stop Hypertension (DASH) diet adherence reported small and statistically uncertain differences in eGFR, with sparse albuminuria data and most evidence derived from a limited number of cohorts using self-reported dietary intake measures [12].

Taken together, this body of evidence supports an individualized approach to hypertension management in CKD, integrating out-of-chair blood pressure assessment, rigorous volume and sodium management, and judicious antihypertensive selection tailored to dialysis timing, comorbidity profile, and symptom burden.

## 3. Pathophysiological Mechanisms

**Figure 1:**
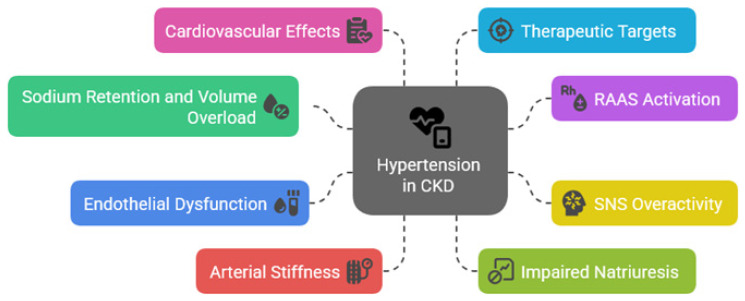
Pathophysiologic mechanisms of hypertension in chronic kidney disease (CKD). RAAS = renin–angiotensin–aldosterone system; SNS = sympathetic nervous system.

Hypertension in patients with CKD arises from a complex interplay of hemodynamic, neurohormonal, and structural abnormalities, with sodium and volume retention due to impaired renal excretory capacity proving as fundamental contributors (Figure 1) [16]. Reduced nephron mass leads to diminished natriuresis, promoting extracellular fluid expansion and increased cardiac output, thereby elevating SBP. This mechanism is particularly relevant in early to moderate stages of CKD, where fluid overload often goes unrecognized (Table 2).

The activation of the RAAS plays a significant role in the hypertensive phenotype of CKD, with declining renal perfusion stimulating renin release, enhancing angiotensin II-mediated vasoconstriction and aldosterone-driven sodium reabsorption [17]. Beyond volume effects, RAAS overactivation contributes to vascular remodeling, myocardial fibrosis, and progressive nephron injury, and thus pharmacologic inhibition of this axis remains a cornerstone in both BP control and renoprotection [18].

Sympathetic nervous system (SNS) overactivity is a hallmark of advanced renal insufficiency and correlates with both hypertension severity and cardiovascular risk. Afferent renal nerve signaling enhances central sympathetic output, exacerbating vasoconstriction, tachycardia, and renin release [19]. This creates a vicious cycle of heightened neurohumoral activation, impaired baroreceptor sensitivity, and refractory hypertension, providing the background for the emerging therapeutic role of RDN, as it can specifically target this mechanism, underscoring its potential value in CKD [20].

Finally, vascular dysfunction contributes significantly to sustained hypertension in renal disease, with endothelial injury, oxidative stress, and arterial stiffness impairing vasodilation and increasing systemic resistance [21]. These processes are compounded by uremic toxins and inflammation, which accelerate arteriosclerosis. Microvascular rarefaction further limits tissue perfusion, perpetuating ischemic injury and hypertensive end-organ damage [22].

## 4. Diagnostic Framework

Accurate diagnosis and classification of hypertension in patients with CKD are foundational to effective management, as hypertension in this context may be categorized as controlled, uncontrolled, or resistant, the latter defined by persistently elevated BP despite the use of three or more antihypertensive agents, including a diuretic [16]. Importantly, these classifications should be interpreted within the context of renal function stage and comorbid conditions. BP targets have been progressively lowered in recent guidelines, with recommendations for a SBP goal of <120 mmHg based on standardized office measurements in patients with CKD and elevated BP, though achieving this safely remains challenging in advanced stages of disease [23].

However, this recommendation contrasts with the 2023 European Society of Hypertension (ESH) guidelines [24], which advise against targeting systolic blood pressure values below 120/70 mmHg in patients with CKD, citing concerns regarding tolerability and safety. These differences reflect distinct methodological approaches and highlight the need for individualized blood pressure targets, particularly in advanced CKD and frail patients.

Most importantly, BP measurement modality significantly impacts diagnosis, as office BP readings, though widely used, often fail to capture true BP burden due to white-coat or masked hypertension [25]. Conversely, ambulatory BP monitoring (ABPM) and home BP monitoring (HBPM) offer superior prognostic value, particularly in identifying nocturnal hypertension and non-dipping patterns, which are prevalent in CKD and associated with adverse outcomes [26]. Accordingly, ABPM remains the gold standard for diagnosing resistant hypertension and should be utilized when available to confirm true resistance and guide therapy [24].

Nevertheless, practical limitations such as reduced availability, patient tolerance, vascular access issues, and altered circadian patterns in advanced CKD and dialysis populations may restrict its routine implementation.

Volume status assessment is another essential diagnostic component, particularly in dialysis-dependent patients, where interdialytic weight gain and ultrafiltration rates influence BP variability [27]. Clinical evaluation should include signs of volume overload, alongside laboratory markers (e.g., natriuretic peptides) and, where possible, bioimpedance analysis, accompanied by concurrent evaluation of target organ damage—such as left ventricular hypertrophy, albuminuria, or hypertensive retinopathy—supports risk stratification and treatment prioritization. Diagnostic clarity not only directs therapeutic intensity but also distinguishes between pharmacologic resistance and pseudo-resistance due to nonadherence or suboptimal measurement [24].

## 5. Therapeutic Strategies

**Figure 2:**
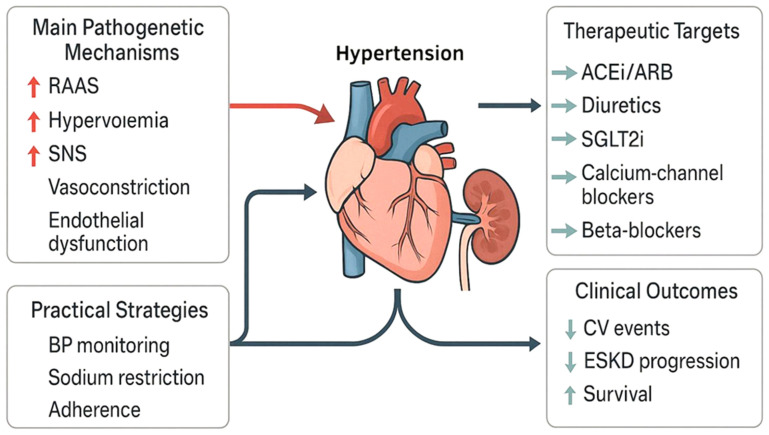
Comprehensive approach to managing hypertension in chronic renal failure. Red arrows indicate major pathogenic mechanisms contributing to hypertension, green arrows represent therapeutic targets and interventions, and blue arrows denote the impact on clinical outcomes. ACEi = angiotensin-converting enzyme inhibitor; ARB = angiotensin receptor blocker; BP = blood pressure; CV = cardiovascular; ESKD = end-stage kidney disease; RAAS = renin–angiotensin–aldosterone system; SGLT2i = sodium-glucose cotransporter-2 inhibitor; SNS = sympathetic nervous system; → = suitable alternatives; ↑ = increased; ↓ = decreased.

Management of hypertension in patients with CKD demands a multifaceted approach that balances effective BP control with renal protection, with overarching goals including minimizing cardiovascular risk, slowing progression to end-stage kidney disease (ESKD), and reducing treatment-related complications (Figure 2) [28]. Guidelines such as those from the Kidney Disease Improving Global Outcomes (KDIGO) Collaborators and the European Society of Hypertension (ESH) emphasize individualized therapy, recognizing the high prevalence of resistant hypertension in this population. Tailored interventions must account for volume status, residual kidney function, comorbidities, and medication tolerance [23,28].

Notably, chlorthalidone has demonstrated significant antihypertensive efficacy even in advanced CKD (stage G4), as shown in the CLICK trial, where it significantly reduced systolic blood pressure compared with placebo, albeit at the cost of increased electrolyte disturbances requiring careful monitoring.

Glucagon-like peptide-1 receptor agonists have also been shown to exert modest blood pressure-lowering effects, alongside established metabolic and renal benefits, as highlighted in dedicated CKD trials such as FLOW.

An overview of pharmacologic and device-based therapeutic options for hypertension in CKD, along with their ideal clinical profiles, is provided in Table 3. Non-pharmacologic strategies remain foundational in CKD-related hypertension. Sodium restriction to less than 2.3 g/day, moderation of fluid intake, weight control, and structured physical activity have demonstrable benefits in lowering BP and enhancing responsiveness to antihypertensive medications (Table 2) [29]. In dialysis patients, achieving and maintaining optimal dry weight through ultrafiltration is critical, while dietary counseling, particularly in potassium and phosphorus management, should align with the CKD stage. In addition, growing emphasis has been placed on patient education and behavioral interventions to improve long-term adherence [30].

**Figure 3:**
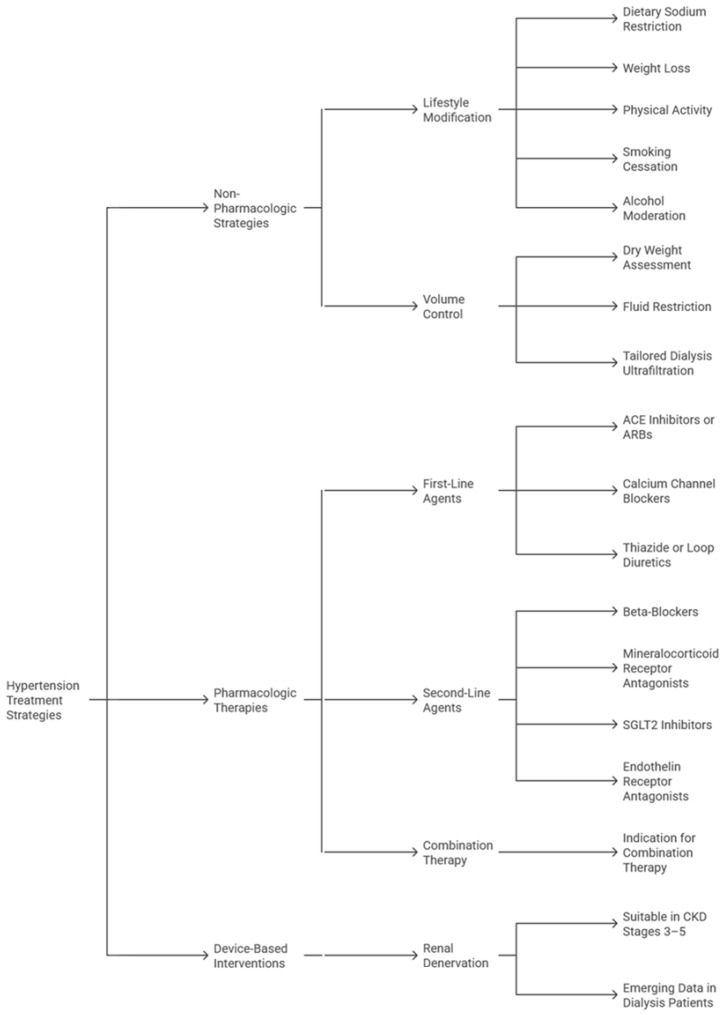
Treatment strategies for hypertension in chronic kidney disease (CKD). ACE = angiotensin-converting enzyme; ARBs = angiotensin receptor blockers; SGLT2 = sodium-glucose cotransporter-2.

Pharmacologic therapy should be individualized based on CKD stage, proteinuria, and cardiovascular risk (Figure 3) [23,24]. Angiotensin-converting enzyme inhibitors (ACEIs) and angiotensin receptor blockers (ARBs) are cornerstone therapies, particularly in proteinuric CKD, owing to their renoprotective and antiproteinuric effects. Diuretics, especially loop diuretics in advanced CKD, play a vital role in volume management. Calcium channel blockers (CCBs) are effective in reducing systolic hypertension and can be safely combined with RAAS inhibitors [31]. Beta-blockers may be considered, particularly in patients with concomitant coronary artery disease or arrhythmia [31]. In resistant hypertension, mineralocorticoid receptor antagonists (MRAs) such as finerenone are increasingly used, although hyperkalemia risk requires close monitoring [32].

Device-based approaches, particularly RDN, offer a compelling therapeutic adjunct for patients with resistant hypertension, a condition frequently observed in CKD [33]. Renal denervation involves catheter-based ablation of sympathetic nerve fibers surrounding the renal arteries, thereby interrupting both efferent and afferent signaling pathways that contribute to hypertension and sympathetic overactivity [4]. The pathophysiological basis is particularly relevant in CKD, where sympathetic hyperactivity is amplified and contributes to progressive renal function decline and heightened cardiovascular risk. A growing body of evidence, including observational studies and small-scale trials, has demonstrated that RDN can safely lower BP in patients with moderate-to-severe CKD, including dialysis-dependent individuals, without causing significant deterioration in renal function [34,35,36].

While these benefits appear durable, with reductions in both office and 24-h ambulatory SBP persisting over months, patient selection remains paramount: ideal candidates are those with documented medication adherence, uncontrolled BP despite optimal pharmacologic therapy, and suitable vascular anatomy for catheter access [37,38]. Indeed, anatomical considerations, such as vascular calcification or altered renal artery structure, are particularly important in CKD patients [39]. While RDN is currently used in select clinical contexts, ongoing randomized trials aim to refine patient eligibility criteria, evaluate long-term safety, and potentially expand its role in routine nephrology and hypertension care.

In summary, successful BP management in renal insufficiency requires integrating pharmacologic and non-pharmacologic therapies with emerging interventional modalities. As evidence accumulates, particularly for therapies like RDN, clinicians should remain agile in updating treatment algorithms. Multidisciplinary collaboration among nephrologists, cardiologists, dietitians, and primary care providers is essential for achieving optimal outcomes in this high-risk population. Precision in tailoring therapy not only controls BP but may also mitigate the accelerated progression of CKD and its cardiovascular sequelae.

## 6. Hypertension in Special Renal Populations

Hypertension is highly prevalent among patients undergoing dialysis, affecting up to 80% of this population, and is multifactorial in nature, driven by volume overload, sodium retention, arterial stiffness, and heightened sympathetic activity [33,40]. Volume control remains the cornerstone of management, yet excessive ultrafiltration can paradoxically induce intradialytic hypotension, and ambulatory and interdialytic blood pressure monitoring are considered useful tools for more accurate blood pressure assessment in dialysis patients, although their routine implementation may be limited by practical constraints [27]. Recent studies also support cautious use of RAAS inhibitors and β-blockers, while newer therapies such as SGLT2 inhibitors are currently under investigation for potential adjunctive roles in selected dialysis populations [41].

Post-transplant hypertension occurs in most recipients and is influenced by immunosuppressive agents (especially calcineurin inhibitors), chronic allograft dysfunction, and residual native kidney function [42]. Management must balance cardiovascular protection with graft preservation, with RAAS blockade frequently favored for its antiproteinuric effects, while requiring close monitoring for hyperkalemia and potential effects on graft perfusion. In addition, mTOR inhibitors, CCBs, and diuretics play variable roles based on the patient’s comorbidity profile and graft function [43].

Older adults with CKD often exhibit isolated systolic hypertension and are at elevated risk of orthostatic hypotension and adverse drug events; thus, individualized blood pressure targets are essential, considering frailty, fall risk, and cognitive impairment [44]. Polypharmacy is common, requiring regular medication reviews, while non-pharmacological interventions such as dietary sodium restriction and supervised exercise programs are underutilized but remain important components of comprehensive management in this group. Overall, tailored and goal-directed management appears to improve clinical outcomes while minimizing treatment burden [45].

## 7. Controversies and Misconceptions

Despite growing awareness of the impact of hypertension in CKD, misconceptions continue to influence clinical decisions and impede optimal management, with a prevalent issue being the overreliance on office BP readings, which may not reflect true BP control due to white-coat or masked hypertension [46]. Ambulatory or home BP monitoring offers superior prognostic value, especially in advanced CKD, where BP variability and nocturnal hypertension are common, yet, unfortunately, these tools are underutilized in nephrology practice, contributing to misclassification and therapeutic inertia [47].

A second enduring myth is the hesitancy to continue RAAS inhibitors in patients with declining eGFR [48]. Although mild increases in serum creatinine are expected with ACE inhibitors or ARBs, these agents confer substantial renal and cardiovascular protection. Inappropriately discontinuing them out of fear of worsening kidney function may accelerate disease progression and expose patients to adverse outcomes. Guidelines recommend continuing RAAS blockade unless hyperkalemia or symptomatic hypotension mandates withdrawal KDIGO [23].

Another common misconception pertains to dialysis-induced hypotension [27]. Although some patients experience intradialytic BP drops, this should not preclude treatment of pre-dialysis hypertension, which is associated with increased mortality. Evidence suggests that volume overload, not dialysis per se, drives most hypertensive phenotypes in this population; individualized dry weight targets and dietary sodium restriction remain pivotal interventions.

Finally, therapeutic inertia—defined as failure to intensify treatment despite uncontrolled BP—remains a critical barrier [49]. Clinicians may mistakenly attribute persistent hypertension to patient nonadherence alone, overlooking structural barriers and biologic resistance. Overcoming this inertia requires structured assessment tools, multidisciplinary input, and consideration of adjunctive strategies such as RDN in carefully selected patients [50].

## 8. Conclusions

Recent advances in hypertension management increasingly emphasize individualized approaches supported by precision medicine and emerging technologies. Biomarker-guided therapy may, in the future, help identify patient subgroups more likely to respond to specific interventions such as renal denervation or novel antihypertensive agents, including SGLT2 inhibitors and aldosterone synthase blockers. Artificial intelligence (AI)-based tools and digital monitoring strategies, particularly wearable devices, are currently under investigation as means to improve blood pressure tracking and treatment adherence, although their routine clinical implementation remains limited. Ongoing and upcoming clinical trials in patients with advanced CKD are expected to clarify the long-term efficacy, safety, and cost-effectiveness of renal denervation, thereby informing its potential role in future therapeutic strategies for resistant hypertension in this population.

## Figures and Tables

**Table 1:** Published systematic reviews on hypertension and chronic kidney disease (CKD). BP = blood pressure; CKD = chronic kidney disease; CV = cardiovascular; DASH = Dietary Approaches to Stop Hypertension; ESKD = end-stage kidney disease; HD = hemodialysis; RR = relative risk; UACR = urine albumin-serum creatinine ratio.

1st Author, Year	Focus	Studies Included	Main Findings	Main Limitations
Aderoju, 2025 [12]	DASH diet adherence in CKD and association with CKD progression markers (eGFR and UACR).	4	eGFR changes were small and not statistically significant (low adherence: +0.54 mL/min/1.73 m^2^; high adherence: +3.34 mL/min/1.73 m^2^). Only 1 study reported UACR; higher adherence corresponded to lower median UACR vs. low adherence.	Key limitations: diet adherence measured via self-report/FFQ, one-time diet assessments, heterogeneity/small number of studies, and only one UACR study; evidence largely from America/Europe with no African studies.
Aftab, 2020 [13]	Ideal systolic BP range/targets in hypertensive ESKD patients on hemodialysis, using mortality as the key outcome.	6	Overall, BP-lowering interventions were associated with reduced all-cause mortality (pooled RR 0.69, 95% CI 0.53–0.90). Authors conclude <140 mmHg systolic is a “promising” target.	Evidence base small; some risk-of-bias concerns. Search limited to English-language articles.
Rabbani, 2022 [15]	BP control strategies in hypertensive hemodialysis patients (pharmacologic context plus volume/dialysis-related approaches; emphasis on dialysis/volume strategies).	12	Summarizes multiple interventions used to control BP in hypertensive HD patients; discusses BP control without antihypertensive drugs (e.g., dialysis/volume strategies) and notes suggested HD BP range (e.g., ~130–160 systolic until stronger data).	Notes important gaps and calls for better study design/reporting and confounder assessment.

**Table 2:** Pathophysiological mechanisms linking hypertension and chronic kidney disease (CKD). CV = cardiovascular; ESKD = end-stage kidney disease; LVH = left ventricular hypertrophy; NO = nitric oxide; RAAS = renin–angiotensin–aldosterone system; RND = renal denervation.; → = casuses; ↑ = increased.

Mechanism	Description	Real-World Example	Prevalence and Impact on Hard Events	Clinical Implication
Sodium and volume overload	Impaired natriuresis → expansion of extracellular volume	Overhydration in dialysis; interdialytic weight gain	~80% in ESKD; associated with LVH, stroke, mortality	Need for aggressive volume and salt control
RAAS activation	Vasoconstriction and aldosterone-driven salt retention	Proteinuric diabetic nephropathy	Present in all CKD stages; linked to progression, CV mortality	Justifies RAAS blockade
Sympathetic nervous system overdrive	Afferent and efferent neural hyperactivity	Elevated norepinephrine in ESKD patients	↑ sympathetic tone in 100% ESKD; ↑ sudden cardiac death risk	Targeted by RDN
Endothelial dysfunction	Reduced NO bioavailability and oxidative stress	Low flow-mediated dilation in CKD patients	Found in ≥60% CKD; predictor of CV events	Encourages statins, anti-inflammatories
Microcirculatory impairment	Capillary rarefaction, hypoxia, impaired autoregulation	Reduced capillary density in renal biopsies	Subclinical; related to ischemic nephropathy, heart failure	Limited therapeutic targeting yet emerging

**Table 3:** Therapeutic approaches for hypertension (HTN) in chronic kidney disease (CKD). ACE = angiotensin-converting enzyme; ARB = angiotensin receptor blocker; BP = blood pressure; CAD = coronary artery disease; CCB = calcium channel blocker; eGFR = estimated glomerular filtration rate; LVH = left ventricular hypertrophy; MRA = mineralocorticoid receptor antagonist; SGLT2 = sodium-glucose cotransporter-2.

Treatment	Purported Benefits	Ideal Candidate	Possible Candidate
ACE inhibitors/ARBs	Reduce proteinuria, slow CKD progression, lower BP	CKD with albuminuria, diabetes, hypertension	Normoalbuminuric CKD with hypertension
Beta-blockers	Reduce heart rate, LVH; useful in ischemia	CKD with CAD or heart failure	Advanced CKD with high sympathetic tone
Calcium Channel Blockers (CCBs)	Effective BP control, salt-insensitive	CKD with isolated systolic hypertension	General CKD patients without albuminuria
Diuretics (Loop/Thiazide)	Volume control, enhanced natriuresis	Volume-expanded CKD stage 3–5, resistant hypertension	Controlled HTN with borderline fluid status
MRAs (e.g., finerenone)	Anti-fibrotic, further RAAS inhibition, proteinuria reduction	Diabetic CKD with persistent albuminuria on ACEI/ARB	Non-diabetic CKD with proteinuria
Renal Denervation	Reduces sympathetic activity and BP, medication sparing	Resistant HTN with eGFR >30, failed triple therapy	Selected ESKD patients on dialysis with refractory BP (investigational use)
SGLT2 inhibitors	Cardioprotective, reduce albuminuria, mild BP reduction	CKD stage 2–4 with diabetes or proteinuria	Non-diabetic CKD with residual albuminuria

## Data Availability

No new data were created or analyzed in this study. Data sharing is not applicable.

## Abbreviations

The following abbreviations are used in this manuscript:

ABPM	Ambulatory blood pressure monitoring
ACEi	Angiotensin-converting enzyme inhibitor
ARB	Angiotensin receptor blocker
BP	Blood pressure
CCB	Calcium channel blocker
CKD	Chronic kidney disease
eGFR	Estimated glomerular filtration rate
ESH	European Society of Hypertension
HBPM	Home blood pressure monitoring
KDIGO	Kidney Disease Improving Global Outcomes
MRA	Mineralocorticoid receptor antagonist
RAAS	Renin–angiotensin–aldosterone system
RDN	Renal denervation
SBP	Systolic blood pressure
SGLT2	Sodium-glucose cotransporter-2
SNS	Sympathetic nervous system
